# Dangers of Blisters

**Published:** 1899-01

**Authors:** 


### DANGERS OF BLISTERS.

Dr. Huchard (*Bull. de l'Acad. de Med.*, No. 7, 1898) expresses the following opinion concerning blisters:

1. They often produce an open wound, which facilitates secondary infections or the absorption of cantharides.
2. Besides tending to cause inflammation of the kidneys and bladder, they have a general congestive action.
3. Even in those diseases where they are most frequently used, such as pneumonia and pleurisy, they should be discarded, because, though they increase pulmonary ventilation, they increase also pulmonary congestion.
4. Blisters tend to arrest excretion by the kidneys, so important in all infectious diseases, and this is especially harmful in those normally causing albuminuria. Instead of aiding the excretion of toxins, blisters are likely to produce a fresh intoxication.
5. The only real use of blisters is in their revulsive and analgesic action, but the effect is better attained by less dangerous means, such as mustard plasters or cold baths.—*Medical Progress.*

